# Digital cognitive behavior therapy for insomnia improving sleep quality: a real-world study

**DOI:** 10.1186/s12888-022-04411-2

**Published:** 2022-12-06

**Authors:** Sugai Liang, Hongjing Mao, Jingyun Yang, Wei Deng, Bo Cao, Zhenghe Yu, Lili Yang, You Xu, Nannan Hu, Wenjuan Liu, Andrew J. Greenshaw, Tao Li

**Affiliations:** 1 Affiliated Mental Health Centre & Hangzhou Seventh People’s Hospital, Zhejiang University School of Medicine, 310013 Hangzhou, Zhejiang China; 2 Department of Neurological Sciences, Rush University Medical Center, 60612 Chicago, IL USA; 3 Department of Psychiatry, University of Alberta, T6G 2B7 Edmonton, AB Canada

**Keywords:** Digital cognitive behavior therapy (dCBT), Insomnia, Depression, Anxiety, Sleep quality

## Abstract

**Background:**

Digital cognitive behavior therapy for insomnia (dCBT-I) is an effective treatment in alleviating insomnia. This study examined the effect of dCBT-I for improving sleep quality in patients with insomnia complaints from a clinical population in a real-world setting.

**Methods:**

The study included 6,002 patients aged 18 years and above with primary complaints of dissatisfying sleep from a sleep clinic in a psychiatric hospital from November 2016 to April 2021. Patients were diagnosed with insomnia, anxiety disorders, or anxiety comorbid with insomnia or depression according to ICD-10. A mobile app was developed for self-reported assessment and delivering dCBT-I interventions and treatment prescriptions to participants. The primary outcome was change in global sleep quality measured by the Pittsburgh Sleep Quality Index (PSQI). At 8- and 12-week follow-up, 509 patients were reassessed. Data were analyzed with non-parametric tests for repeated measures.

**Results:**

Patients treated with dCBT-I monotherapy were younger, with a more frequent family history of insomnia compared to those with medication monotherapy and those with combined dCBT-I and medication therapy. Improvements of sleep quality from baseline to 8-week follow-up were significant in each treatment type. Compared to 8-week follow-up, PSQI scores at 12-week were significantly decreased in the depression group receiving combined therapy and in the anxiety group treated with dCBT-I monotherapy and with combined therapy. A time-by-treatment interaction was detected in anxiety patients indicating differential reduction in PSQI scores over time between different treatment options.

**Conclusion:**

The current findings suggest dCBT-I is a practical and effective approach for lessening insomnia symptoms, especially for patients with anxiety symptoms suggesting with a more extended intervention period (i.e., 12 weeks).

**Trial registration:**

Chinese Clinical Trial Registry (ChiCTR1900022699).

**Supplementary Information:**

The online version contains supplementary material available at 10.1186/s12888-022-04411-2.

## Background

Insomnia, which affects 10 to 15% of the general population [1], is characterized by dissatisfaction with sleep quality or duration and difficulty initiating or maintaining sleep, and is accompanied by daytime functioning deficits [2, 3]. Insomnia has several detrimental consequences, including fatigue, low work productivity, and reduced neurocognitive functions [4]. Additionally, insomnia is a risk factor for major depressive disorder, anxiety disorders, substance use problems, hypertension, diabetes and other morbidities [5]. A bidirectional relationship may exist between insomnia, anxiety and depression, whereby insomnia may predict and be predicted by anxiety and depression [6]. In addition, sleep disturbances influencing global functioning are associated with increased risk for cause-specific work disability, subsequent disabling mental disorders and various physical illnesses [7, 8]. Persistent insomnia over six years is associated with increased risk for all-cause and cardiopulmonary mortality relative to intermittent insomnia or lack of insomnia [2, 9]. Given the severe health consequences of insomnia, there is an urgent need for feasible, acceptable and effective interventions to alleviate insomnia symptoms.

In this context, cognitive behavioral therapy for insomnia (CBT-I) is considered the first-line therapy for all patients with insomnia, including those with coexisting conditions [2, 10, 11]. CBT-I is a directive, sleep-focused and time-limited psychotherapeutic method designed to modify behavioral and thinking patterns that are presumed to exacerbate or perpetuate insomnia [2, 10]. CBT-I is equally effective for treating insomnia in the short term, but its effect is more durable than medications [2, 12]. With recent developments in internet technology, an innovative and interactive solution has been developed to deliver digital CBT-I (dCBT-I) via online platforms and digital media. dCBT-I is non-inferior ineffectiveness compared to face-to-face delivery of CBT [13–16], and it is remotely and rapidly accessible [17]. In a randomized controlled trial, we found that dCBT-I is as effective as face-to-face individual CBT-I in decreasing sleep onset latency and improving sleep efficiency in patients with insomnia, but dCBT-I is superior in decreasing use of sleep medication and improving daytime function [18]. Additionally, clinical studies indicate that dCBT-I is effective in decreasing insomnia severity and improving sleep quality in insomnia and other comorbid mental disorders [17, 19, 20].

In real-world settings, it remains unclear how effective dCBT-I may be in patients with a chief complaint of insomnia or dissatisfaction with sleep quality, assessed by a sleep clinic in a psychiatric hospital. The current study focused on patients diagnosed with insomnia, or insomnia comorbid with anxiety, or with depression or anxiety disorders, which are the most common insomnia-related mental health complaints in the clinic. Participating patients via a mobile app reported self-assessment measures and current treatments, which comprised dCBT-I monotherapy, medication alone, combined medication and dCBT-I or no treatments. This study aimed (1) to investigate the demographic and clinical characteristics and treatments of individuals with insomnia complaints across different diagnostic categories, (2) to examine the relative efficacy of dCBT-I monotherapy and other treatments between baseline and 8-week and 12-week follow-up time points in respective insomnia, anxiety and depression groups.

## Methods

### Data source

This retrospective observational study examined clinical data from a mobile app named *Good Sleep 365 Days* which was developed to record demographic information, perform self-reported assessments and deliver programmed dCBT-I and professional consulting. The present study was carried out in the Sleep Health Project in Zhejiang Province, China. 7,223 patients with a chief complaint of insomnia or dissatisfaction with sleep quality were recruited from a sleep clinic. All subjects provided online informed consent before participation. This study was approved by the local research ethics committee in accord with the Declaration of Helsinki and registered at the Chinese Clinical Trial Registry (ChiCTR1900022699).

### Study design

**Fig. 1:**
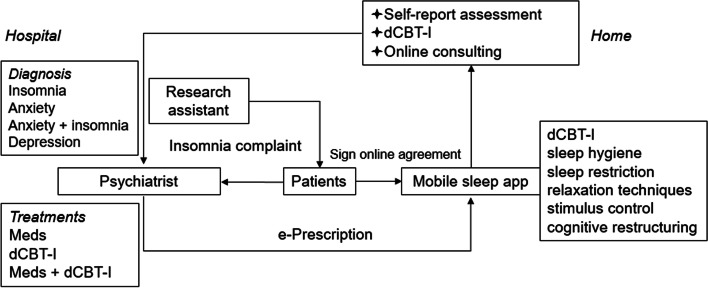
Patient visit process. Participants were trained individually on how to use the app by a research assistant. Patients were included in this study if they were interviewed with a psychiatrist and diagnosed with insomnia, anxiety disorders, or anxiety comorbid with insomnia or depression. Treatment plans were delivered to patients via the mobile app

Using a retrospective design, data were collected from November 2016 to April 2021. Figure 1 schematically illustrates the patient visit process. In the prediagnosis step, participants were trained on how to use the app by a research assistant in a clinic waiting room. Then they were interviewed by experienced psychiatrists. Participants were eligible for inclusion if they were above 18 years of age, and diagnosed with insomnia, anxiety disorders, or anxiety comorbid with insomnia or depression according to criteria of the International Classification of Diseases Tenth Revision (ICD-10), and fully completed the baseline demographic information. Demographic information including age, sex, educational level and employment status was recorded. Clinical data collected included illness duration, family history of insomnia, history of taking psychotropic medications, the effect of stressful life events and current treatments. The question for family history of insomnia was: “Do any of your immediate family members presently have or ever had sleep difficulties?” A family history of insomnia was defined as a report of at least one first-degree, biological relative (sibling, child, or parent) with past or current insomnia.

The mobile app has a series of validated and established patient self-report instruments. The Pittsburgh Sleep Quality Index (PSQI) was used to assess the multifactorial construct of self-perceived sleep quality. The Epworth Sleepiness Scale (ESS) was used to measure each participant’s daytime sleepiness. The severity of depression and anxiety were assessed using the Patient Health Questionnaire (PHQ-9) and the Generalized Anxiety Disorder 7-Item Scale (GAD-7), respectively. The Patient Health Questionnaire Somatic Symptom Severity Scale (PHQ-15) was used to assess somatic symptom severity. The components of dCBT-I include sleep hygiene, sleep restriction, relaxation techniques, stimulus control and cognitive restructuring [21]. The app had videos of each training session. See Supplementary Materials for further details. Homework was assigned to participants to allow time to practice these skills between sessions. Messages were automatically sent via the app to remind participants to complete the tasks. The treatment effect for insomnia was reassessed via the mobile app. dCBT-I consisted of 8 weekly sessions, and the cognitive and behavioral components were assigned appropriately based on online self-assessment reports during 8-week to 12-week follow-up measures.

### Primary outcomes

Total PSQI scores were assessed as primary outcomes. Supplementary Fig. 1 represents the steps for data screening. A final set of 6,002 participants was included in the analysis. A subset of individuals had data available at both 8- and 12-week follow-up. 509 participants completed the self-report PSQI scale.

### Statistical analysis

We performed statistical analysis by using chi-square tests for categorical variables (sex, educational level, employment status and family history of insomnia), analysis of variance (ANOVA) for age and non-parametric Kruskal-Wallis tests for other clinical characteristics between patient groups. Data distribution was assessed using Shapiro-Wilk test, and *P* < .05 indicated that a variable was not normally distributed. The non-parametric Friedman test for repeated measures was used to compare total PSQI scores assessed at baseline, 8-week, and 12-week follow-up. We used G*Power v3.1.9 to estimate the sample size required. We found that with 3 observation times among 1 group and projected medium effect size (*d* = 0.30), more than 19 for an ANOVA with repeated measures are needed with α = 0.05. Consequently, we adopted a sample size for the treatment group of n ≥ 19 for further analysis.

For post hoc analyses, Wilcoxon signed-rank tests were used for non-parametric paired comparisons, and Wilcoxon rank-sum tests were used for unpaired experimental measures. The two-way Wald-type statistic (*WTS)* was conducted to compare differences in primary outcomes for the factors of time and treatments using the R package *nparLD* [22]. *Relative treatment effect (RTE)* was reported as a measure of effect size. *RTE* represents a probability that a randomly drawn observation from a particular group has a more significant value than that from the combined mean distribution [23]. *RTE* is a probability value between 0 and 1, with 0.5 signifying no effect and 0 and 1 signifying complete separation of the two groups. P values were corrected for multiple comparisons using the false discovery rate (FDR) procedure, and *P*_*FDR*_ < 0.05 were declared to be statistically significant. Python 3.6.5 and R version 3.6.1 were used to perform the analyses.

## Results

### Characteristics of participants

**Fig. 2:**
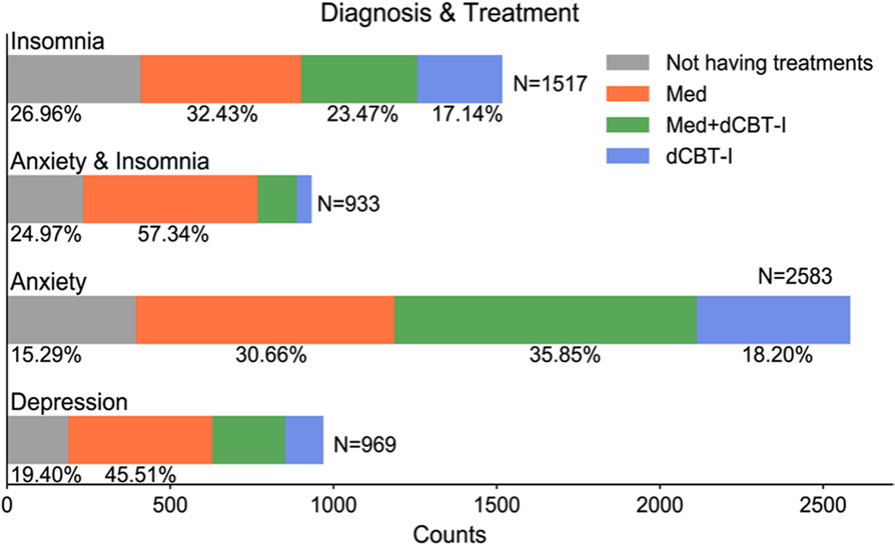
Percentage of patients with insomnia complaints in each treatment option. Med, medication. dCBT-I, digital cognitive behavior therapy for insomnia

Overall, 6,002 participants were included, with 1,517 [25.27%] in the insomnia group, 2,583 [43.05%] in the anxiety disorders group, 933 [15.54%] in the anxiety disorders comorbid with insomnia group and 969 [16.14%] in the depression group. Table 1 presents baseline demographic and clinical characteristics for patients with the chief complaint of unsatisfied sleep quality by specific diagnostic categories. Those four diagnostic groups exhibited statistically significant differences in demographic and clinical variables. Figure 2 shows different treatments in each diagnostic group. 19.40–26.96% of patients with insomnia complaints reported not having treatments.

**Table 1:** Demographic and clinical characteristics of participants with insomnia complaints

Variables	Insomnia	Anxiety & Insomnia	Depression	Anxiety	Statistics
	(*n* = 1517)	(*n* = 933)	(*n* = 969)	(*n* = 2583)	*χ² / F / H*
Age (years)	44.16 (13.21)	46.88 (11.72)	41.51 (14.46)	44.79 (12.11)	29.40**
Sex (Female / Male)	982 (64.73%) / 535	707 (75.78%) / 226	697 (71.93%) / 272	1925 (74.53%) / 658	52.98**
Educational Level (low / medium / high / unknown)	519 / 257 / 733 / 8	401 / 156 / 373 / 3	338 / 179 / 443 / 9	1120 / 467 / 965 / 31	61.87**
Employment status (Yes / No / unknown)	769 / 395 / 353	513 / 377 / 43	459 / 231 / 279	1176 / 600 / 807	22.63**
Duration (< 3 months / 3–12 months / > 12 months)	389 / 276 / 852	190 / 172 / 571	357 / 210 / 402	688 / 430 / 1465	101.75**
Family history of insomnia (yes / no / unknown)	453 / 1063 / 1	283 / 650 / 0	323 / 645 / 1	926 / 1656 / 1	19.31**
First episode (yes / no / unknown)	316 / 1107 / 94	222 / 682 / 29	240 / 628 / 101	507 / 1695 / 381	9.90*
History of psychotropic medication (yes / no / unknown)	750 / 766 / 1	539 / 394 / 0	552 / 416 / 1	1460 / 1122 / 1	25.63**
Med / dCBT-I / Combined therapy / Not having treatments	492 / 260 / 356 / 409	535 / 45 / 120 / 233	441 / 117 / 223 / 188	792 / 470 / 926 / 395	501.10**
Impact of Life Events	497 / 7.00 (5.00)	657 / 7.00 (5.00)	293 / 8.00 (5.00)	665 / 8.00 (5.00)	29.39**
Pittsburgh Sleep Quality Index	1110 / 14.00 (6.00)	342 / 16.00 (5.00)	750 / 16.00 (6.00)	2065 / 15.00 (6.00)	58.51**
GAD-7	861 / 3.00 (5.00)	111 / 6.00 (6.00)	602 / 13.00 (11.00)	1769 / 6.00 (8.00)	557.44**
PHQ-9	865 / 4.00 (4.00)	112 / 6.00 (7.00)	607 / 17.00 (10.00)	1778 / 7.00 (7.00)	647.09**
PHQ-15	1052 / 6.00 (4.00)	308 / 8.00 (5.00)	717 / 11.00 (7.00)	1998 / 9.00 (6.00)	443.18**
Epworth Sleepiness Scale	892 / 3.00 (5.00)	139 / 4.00 (5.50)	640 / 5.00 (7.00)	1838 / 4.00 (5.00)	37.67**

Mean and standard deviation (SD) for age. A total of number of subjects performed self-reported scales, and median and interquartile range (IQR) of scale scores at baseline are listed. Med, medication. dCBT-I, digital Cognitive Behavior Therapy for insomnia. Combined therapy, medication and dCBT-I. GAD-7, Generalized Anxiety Disorder 7-Item Scale. PHQ-9, Patient Health Questionnaire-9. PHQ-15, Patient Health Questionnaire Somatic Symptom Severity Scale. Impact of life events on insomnia was scored from 0 (not at all) to 10 (very serious). For educational level, low level represented middle school education and less, medium level indicated parts of or completed high school level or high school training, and high level represented college degree and above

* *P* < .05. ** *P* < .001

In the insomnia group, 492 [32.43%] patients had medication alone, 260 [17.14%] patients had dCBT-I monotherapy, 356 [23.47%] had combined therapy, and 409 [26.96%] patients reported not having any treatment. See Supplementary Table 1 for characteristics of different treatment types. Compared to patients with insomnia treated with medication monotherapy and with combined therapy, patients with dCBT-I monotherapy were relatively younger [Mean age 42.19 years, standard deviation (SD) 11.85] (*d* = 0.30, *P*_*FDR*_ < 0.00; *d* = 0.33, *P*_*FDR*_ < 0.001, respectively) with a higher percentage of employment status [69.52%] and family history of insomnia [36.15%], and a lower percentage of long illness duration (> 12 months) [52.31%] and history of taking psychotropic medications [25.38%], and lower PSQI scores (*d* = 0.57, *P*_*FDR*_ < 0.001; *d* = 0.62, *P*_*FDR*_ < 0.001, respectively). The descriptive statistics of variables between different treatment options are listed in Supplementary Tables 2, 3 and 4 for anxiety disorders group, anxiety comorbid with insomnia group, and depression group, respectively. See supplementary results for more details.

### Effects of dCBT-I on sleep quality in the follow-up subset

For the primary outcome - PSQI scores, 509 patients had data available at 8- and 12-week follow-up, with 97 in the insomnia group, 298 in the anxiety group, 46 in the anxiety comorbid with insomnia and 68 in the depression group. Table 2 lists the variables of different treatment options with sample size ≥ 19.

**Table 2:** Demographic and clinical characteristics of longitudinal subset

Variables	Insomnia		Anxiety & Insomnia	Anxiety			Depression
	dCBT-I	Med + dCBT-I	Med + dCBT-I	Med	dCBT-I	Med + dCBT-I	Med + dCBT-I
	(*n* = 24)	(*n* = 65)	(*n* = 27)	(*n* = 19)	(*n *= 81)	(*n* = 189)	(*n* = 46)
Age (years)	47.83 (13.08)	50.35 (10.98)	46.44 (10.71)	49.26 (12.89)	46.48 (10.12)	47.98 (9.50)	46.59 (10.52)
Sex (Female / Male)	16 / 8	44 / 21	21 / 6	16 / 3	64 / 17	144 / 45	33 / 13
Educational Level (low / medium / high / unknown)	10 / 5 / 9	20 / 14 / 31	8 / 6 / 13	8 / 6 / 4	38 / 21 / 21	77 / 44 / 64	14 / 12 / 20
Employment status (Yes / No / unknown)	11 / 9 / 4	36 / 18 / 11	13 / 13 / 1	7 / 6 / 6	43 / 17 / 21	102 / 48 / 39	29 / 10 / 7
Duration (< 3 months / 3–12 months / > 12 months)	5 / 3 / 16	12 / 11 / 42	8 / 6 / 13	4 / 3 / 12	23 / 11 / 47	39 / 37 / 113	16 / 8 / 22
Family history of insomnia (yes / no)	10 / 14	18 / 47	10 / 17	10 / 9	36 / 45	64 / 125	20 / 26
First episode (yes / no / unknown)	7 / 13 / 4	15 / 48 / 2	9 / 16 / 2	6 / 12 / 1	15 / 35 / 31	47 / 118 / 24	10 / 33 / 3
History of psychotropic medication (yes / no)	8 / 16	43 / 22	20 / 7	14 / 5	25 / 56	124 / 65	36 / 10
PSQI baseline	14.50 (2.50)	15.00 (5.00)	16.00 (4.00)	17.00 (5.50)	15.00 (6.00)	16.00 (7.00)	18.00 (6.75)
PSQI 8 weeks	8.50 (4.25)	9.00 (5.00)	9.00 (4.00)	10.00 (5.00)	9.00 (6.00)	10.00 (4.00)	10.00 (5.00)
PSQI 12 weeks	8.00 (4.00)	9.00 (3.00)	9.00 (5.00)	8.00 (5.50)	9.00 (6.00)	10.00 (6.00)	9.50 (6.00)
Baseline vs.8 weeks	7.50**	17.50**	0.00**	2.00**	378.00**	1005.00**	61.00**
Baseline vs. 12 weeks	8.50**	8.00**	8.00**	2.00**	237.50**	1005.00**	17.00**
8 weeks vs. 12 weeks	87.00	503.00	95.00	34.50	558.00*	4559.50**	235.00*

Mean and standard deviation (SD) for age. Median and Interquartile range (IQR) for PSQI scores. *Med *Medication, *dCBT-I *Digital Cognitive Behavior Therapy for insomnia, *PSQI* Pittsburgh Sleep Quality Index. Wilcoxon signed-rank tests for non-parametric paired comparisons. W value is listed. * *P*_*FDR*_ < 0.05. ** *P*_*FDR*_ < 0.001

**Fig. 3:**
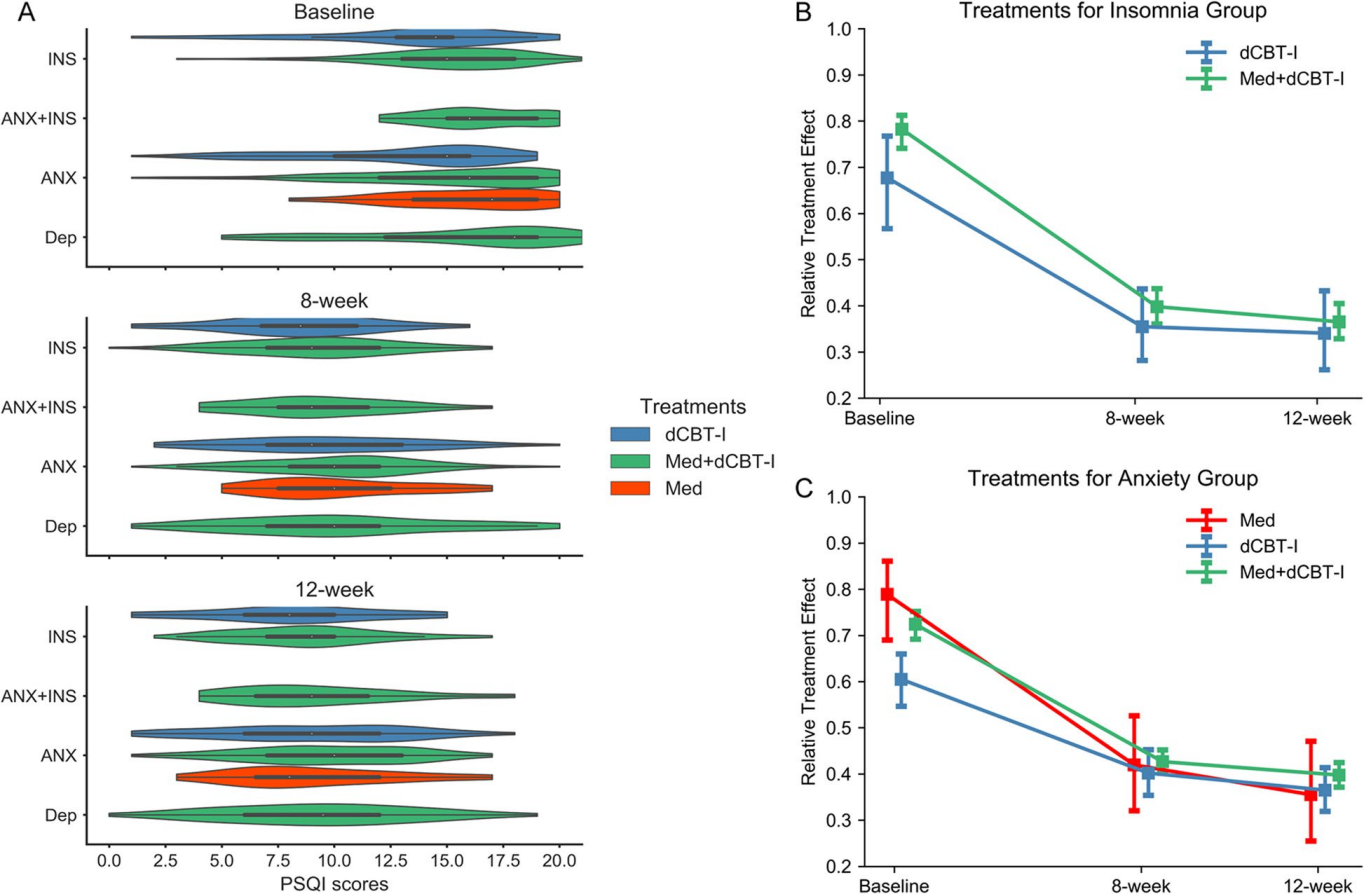
Comparisons of PSQI scores at baseline and follow-up. **A** Violin plot of PSQI scores for each treatment in different clinical diagnoses at baseline and 8-week and 12-week follow-up. INS, insomnia group. ANX, anxiety disorder group. INS + ANX, insomnia comorbid with anxiety disorder group. Dep, depression group. **B** relative treatment effect (RTE) of dCBT-I (digital cognitive behavior therapy for insomnia) monotherapy and combined therapy in the insomnia group at baseline and 8-week and 12-week follow-up. **C** RTE of dCBT-I monotherapy, medication monotherapy and combined therapy in the anxiety disorders group at baseline, 8-week and 12-week follow-up

In the insomnia group treated with dCBT-I monotherapy, reduced PSQI scores were detected at 8- and 12-week follow-up compared to baseline (*P*_*FDR*_ < 0.001, see Table 2), but no significant differences were found in PSQI scores between 8- and 12-week follow-up (*P*_*FDR*_ = 0.51). Similar changes in PSQI scores were observed between baseline and 8- and 12-week follow-up in the insomnia group with combined therapy and the anxiety comorbid with insomnia group with combined therapy. In Fig. 3A, a violin plot represents the distribution of PSQI scores for different treatment types at baseline and follow-up.

In patients diagnosed with anxiety disorders, significantly lower PSQI scores were achieved in each treatment group at 8- and 12-week follow-up compared to baseline (*P*_*FDR*_ < 0.001). The further reduction of PSQI scores were detected at 12-week compared to 8-week follow-up in patients treated with CBT-I monotherapy (*P*_*FDR*_ = 0.03) and those with combined therapy (*P*_*FDR*_ = 0.01), but not in those receiving medication alone (*P*_*FDR*_ = 0.15). Similarly, reduced PSQI scores were detected in the 12-week compared to 8-week follow-up in the depression group receiving combined therapy.

### Relative treatment effect between treatment and time

In the insomnia group, for the primary outcome - PSQI scores, the main effect of time was significant (*WTS* = 151.18, *P*_*FDR*_ < 0.001). At baseline, 8- and 12-week follow-up, *RTEs* = 0.68, 0.35, 0.34 for dCBT-I monotherapy and *RTEs* = 0.78, 0.40, 0.37 for combined therapy. The main effect of time was significant different in PSQI scores between patients treated with CBT-I monotherapy and those with combined therapy (*WTS* = 100.15, *P*_*FDR*_ < 0.001, *RTEs* = 0.46, 0.52 for these two treatments). Figure 3B represents rapid improvement observed for both treatment options with a significant time effect. Significant decreases of PSQI scores at 8-week follow-up compared to baseline indicated fewer sleep problems and better sleep quality. However, there were no statistically significant differences in the PSQI scores at 8- and 12-week follow-up time points in the insomnia group with each treatment type. Neither the main effect of treatment options nor the treatment-by-time interaction were significant in the insomnia group (*WTS* = 1.61, *P*_*FDR*_ = 0.41; *WTS* = 1.42, *P*_*FDR*_ = 0.49, respectively).

In the anxiety group, the main effect of time was significant in the PSQI scores (*WTS* = 193.12, *P*_*FDR*_ < 0.001, at baseline, 8- and 12-week follow-up RTEs = 0.79, 0.42, 0.36 for medication alone, *RTEs* = 0.61, 0.40, 0.37 for CBT-I monotherapy, and *RTEs* = 0.72, 0.43, 0.40 for combined therapy). The significant main effect of time in PSQI scores was different between each two treatment types (*P*_*FDR*_ < 0.001). Significant treatment-by-time interaction was observed (*WTS* = 11.05, *P*_*FDR*_ = 0.04). The treatment-by-time interaction in the dCBT-I monotherapy group was significantly different from that of the medication alone group (*P*_*FDR*_ = 0.02), and that of the combined therapy group (*P*_*FDR*_ = 0.02). The significant interaction effect indicates that the plots of PSQI scores are not parallel over the time points between the two treatment types. See Fig. 3C. However, there were no significant differences in the treatment-by-time interaction between medication alone and combined therapy (*WTS* = 1.65, *P*_*FDR*_ = 0.20). No main effect of treatment types was detected in the anxiety group (*WTS* = 4.25, *P*_*FDR*_ = 0.12).

## Discussion

To our best knowledge, this is the most extensive real-world study to evaluate the efficacy of dCBT-I delivered via a mobile app for adults with insomnia in a single clinical setting. Our results indicated clinically meaningful reductions in PSQI scores relative to baseline in each diagnostic group with different treatment options. The current findings confirmed the efficacy of dCBT-I for improving subjective feelings on insufficient sleep in patients with insomnia, depression and anxiety disorders. Significant improvements in sleep quality at 12-week compared to 8-week follow-up were observed in the depression group receiving combined therapy and in the anxiety group treated with dCBT-I monotherapy and with combined therapy. This study was also identified the time-by-treatment interaction in the anxiety group manifesting PSQI scores differed between dCBT-I monotherapy and other treatment types over time. Notably, about one-quarter of patients with insomnia complaints did not receive the recommended treatment, which probably contributed to poor prognosis and disease progression of the disorder.

In this study, sleep quality improvement was prominent at 8-week follow-up compared to baseline in the insomnia group treated with dCBT-I monotherapy and with combined therapy, but no significant improvement was observed from 8-week to 12-week follow-up. In the anxiety group, patients receiving medication alone showed a negligible decrease in PSQI scores at 12-week follow-up compared to 8-week, whereas patients treated with combined therapy and with dCBT-I monotherapy exhibited a significant reduction in PSQI scores. Our findings suggested that the effect of dCBT-I was dissimilar to the impact of medication on the improvement of sleep quality. Previous studies have supported the endorsement CBT-I as the best available intervention for patients with insomnia, including those with coexisting mental disorders [11, 19, 20, 24]. Consistent with prior research, the current results indicate that CBT-I may be highly effective in reducing insomnia and improving sleep across a wide array of clinical populations in a real-world setting [5, 19, 24, 25]. In addition, as insomnia and anxiety disorders commonly co-occur, treatment of insomnia may prevent the exacerbation of comorbid anxiety symptoms [5, 6]. A prior study demonstrated that patients with a longer treatment duration of dCBT-I exhibited more significant improvements in reducing insomnia [14]. This study also suggested that 12-week dCBT-I compared to 8-week treatment duration could have more improvements of sleep quality in patients with anxiety. Overall, the current findings highlight the need to increase our understanding of the importance of dCBT-I in treating insomnia symptoms, especially in patients with anxiety disorders.

In this study, individuals with insomnia complaint treated with dCBT-I monotherapy had a higher percentage of family history of insomnia compared to individuals receiving other treatment options in respective insomnia, anxiety, and depression groups. Previous studies have reported that insomnia is heritable and tends to aggregate in families [26–28]. More than a third of individuals with an insomnia complaint have a first- or second-degree relative with a current or past sleep problem which indicates the existence of a familial vulnerability to insomnia [27]. Family and personal history of insomnia are considered as predisposing factors for insomnia [28]. Notably, CBT-I effectively prevents the onset of insomnia, offsetting vulnerability factors and improving functional outcomes in adolescents with a family history of insomnia [29]. In line with previous findings, the current results support the status of CBT-I as a significant intervention for patients with insomnia complaints with a family history of insomnia. Additionally, CBT-I delivered in general primary care effectively improves sleep onset and maintenance in patients with chronic insomnia [30]. Due to accelerating technological improvements, dCBT-I can be an increasingly cost-effective and highly accessible intervention to improve sleep efficiency [31].

Consistent with prior studies, the current findings indicate that dCBT-I monotherapy, medication alone and combined therapy have significant effects on improving sleep quality for patients with insomnia complaints in a short term [2, 10]. Psychological and pharmacological therapies have complementary roles in the treatment of insomnia, whereas CBT-I may result in sustained long-term benefits for patients with insomnia [2, 15, 25, 32]. CBT-I is essential to alter factors that exacerbate or perpetuate insomnia [11, 12, 21]. In addition, dCBT-I is as clinically effective as conventional CBT-I, with equal or superior efficacy to hypnotics but fewer side effects and longer-lasting benefits following the discontinuation of treatment [17, 25]. Moreover, digital health applications represent a promising direction to reduce barriers and problems of delivering adequate treatments for sleep problems. As a potential therapeutic advantage, the prolonged beneficial effect of dCBT-I for insomnia symptoms should be investigated further in future research work.

Although the strengths of our analysis included a large clinical population over several years in a real-world study, several limitations must be acknowledged. First, we did not take into account the physical health status of patients in this study. The association between sleep and physical health is complex, and it is difficult to interpret the effects of health adjustments and separate insomnia from comorbid conditions. Second, this study focused on sleep quality in patients with insomnia complaints and self-reported measures for sleep disturbances and mood disorders. In future studies, it will be advantageous to include evaluation for insomnia severity and objective assessment measures, including polysomnography and actigraphy. Third, participants in the current study were recruited from a sleep clinic in a psychiatric hospital. The future study combined with participants recruited from a general hospital may make the findings more comprehensive. Fourth, this study did not account for socioeconomic status, social media use and physical exercise. In this regard, considering the accessibility of mobile phones, higher social media use at bedtime is associated with poorer sleep outcomes, including shorter sleep duration and poorer sleep quality [33]. Fifth, an unexplored possibility for this study that is relevant for some of these study limitations is the possibility of including include digital technologies such as fitness trackers, augmented reality or virtual reality digital health tools in interventions to combat insomnia. Sixth, attrition rate was relatively high in this real-world study, but comparable to other smartphone apps real-world research [34]. Strategies to improve treatment engagement should be considered, including providing person-to-person feedback, tailoring messages to maintain users’ interests, and incentivizing the adherence to treatment [35, 36].

## Conclusion

Via a large clinical population in a real-world setting, this study found that dCBT-I was effective and accessible for improving sleep quality for patients with insomnia complaints, especially those younger and with a family history of insomnia. The current findings also highlight the need to increase our understanding of the importance of dCBT-I for reducing insomnia in patients with anxiety disorders, as longer treatment duration resulted in more remarkable symptom improvement. Our findings provide the evidence for dCBT-I efficacy as mono- or combined therapy in insomnia, and treatment for helping to guide personalization of treatment in clinical practice.

## Supplementary Information

**Additional file 1.**

## Data Availability

The authors declare that all data supporting the findings of this study are available within the paper and its supplementary materials.

## References

[1] Ohayon MM (2002). Epidemiology of insomnia: what we know and what we still need to learn. Sleep Med Rev.

[2] Morin CM, Drake CL, Harvey AG, Krystal AD, Manber R, Riemann D, Spiegelhalder K (2015). Insomnia disorder. Nat Rev Dis Primers.

[3] Ohayon MM, Reynolds CF (2009). 3rd: epidemiological and clinical relevance of insomnia diagnosis algorithms according to the DSM-IV and the International classification of Sleep Disorders (ICSD). Sleep Med.

[4] International Classification of Sleep Disorders: Diagnostic and Coding Manual. Westchester: American Academy of Sleep Medicine; 2005. p. 51–55.

[5] Krystal AD, Prather AA, Ashbrook LH (2019). The assessment and management of insomnia: an update. World Psychiatry.

[6] Alvaro PK, Roberts RM, Harris JK (2013). A systematic review assessing bidirectionality between sleep disturbances, anxiety, and Depression. Sleep.

[7] Lallukka T, Haaramo P, Lahelma E, Rahkonen O (2011). Sleep problems and disability retirement: a register-based follow-up study. Am J Epidemiol.

[8] Salo P, Oksanen T, Sivertsen B, Hall M, Pentti J, Virtanen M, Vahtera J, Kivimäki M (2010). Sleep disturbances as a predictor of cause-specific work disability and delayed return to work. Sleep.

[9] Parthasarathy S, Vasquez MM, Halonen M, Bootzin R, Quan SF, Martinez FD, Guerra S (2015). Persistent insomnia is associated with mortality risk. Am J Med.

[10] Winkelman JW (2015). CLINICAL PRACTICE. Insomnia disorder. N Engl J Med.

[11] Kallestad H, Vedaa Ø, Scott J, Morken G, Pallesen S, Harvey AG, Gehrman P, Thorndike F, Ritterband L, Stiles TC (2018). Overcoming insomnia: protocol for a large-scale randomised controlled trial of online cognitive behaviour therapy for insomnia compared with online patient education about sleep. BMJ open.

[12] Mitchell MD, Gehrman P, Perlis M, Umscheid CA (2012). Comparative effectiveness of cognitive behavioral therapy for insomnia: a systematic review. BMC Fam Pract.

[13] Ritterband LM, Thorndike FP, Gonder-Frederick LA, Magee JC, Bailey ET, Saylor DK, Morin CM (2009). Efficacy of an internet-based behavioral intervention for adults with insomnia. Arch Gen Psychiatry.

[14] Zachariae R, Lyby MS, Ritterband LM, O’Toole MS (2016). Efficacy of internet-delivered cognitive-behavioral therapy for insomnia - A systematic review and meta-analysis of randomized controlled trials. Sleep Med Rev.

[15] Vedaa Ø, Kallestad H, Scott J, Smith ORF, Pallesen S, Morken G, Langsrud K, Gehrman P, Thorndike FP, Ritterband LM (2020). Effects of digital cognitive behavioural therapy for insomnia on insomnia severity: a large-scale randomised controlled trial. Lancet Digit Health.

[16] Soh HL, Ho RC, Ho CS, Tam WW (2020). Efficacy of digital cognitive behavioural therapy for insomnia: a meta-analysis of randomised controlled trials. Sleep Med.

[17] Luik AI, Kyle SD, Espie CA (2017). Digital cognitive behavioral therapy (dCBT) for Insomnia: a state-of-the-Science Review. Curr Sleep Med Rep.

[18] Mao HJ, Xu Y, Yu ZH (2017). Efficacy of digital delivery of cognitive behavioral therapy for insomnia: a randomized controlled study. Chin J Psychiatry.

[19] Ye YY, Zhang YF, Chen J, Liu J, Li XJ, Liu YZ, Lang Y, Lin L, Yang XJ, Jiang XJ (2015). Internet-based cognitive behavioral therapy for Insomnia (ICBT-i) improves comorbid anxiety and Depression-A Meta-analysis of Randomized controlled trials. PLoS ONE.

[20] Ramsawh HJ, Bomyea J, Stein MB, Cissell SH, Lang AJ (2016). Sleep quality improvement during cognitive behavioral therapy for anxiety Disorders. Behav Sleep Med.

[21] Pigeon WR (2010). Treatment of adult insomnia with cognitive-behavioral therapy. J Clin Psychol.

[22] Noguchi K, Gel YR, Brunner E, Konietschke F (2012). nparLD: an R Software Package for the nonparametric analysis of Longitudinal Data in Factorial experiments. J Stat Softw.

[23] Brunner E, Konietschke F, Pauly M, Puri ML (2017). Rank-based procedures in factorial designs: hypotheses about non‐parametric treatment effects. J R Stat Soc Series B Stat Methodol.

[24] Taylor DJ, Pruiksma KE (2014). Cognitive and behavioural therapy for insomnia (CBT-I) in psychiatric populations: a systematic review. Int Rev Psychiatry.

[25] Morin CM, Vallières A, Guay B, Ivers H, Savard J, Mérette C, Bastien C, Baillargeon L (2009). Cognitive behavioral therapy, singly and combined with medication, for persistent insomnia: a randomized controlled trial. JAMA.

[26] Harvey CJ, Gehrman P, Espie CA (2014). Who is predisposed to insomnia: a review of familial aggregation, stress-reactivity, personality and coping style. Sleep Med Rev.

[27] Bastien CH, Morin CM (2000). Familial incidence of insomnia. J Sleep Res.

[28] Beaulieu-Bonneau S, LeBlanc M, Mérette C, Dauvilliers Y, Morin CM (2007). Family history of insomnia in a population-based sample. Sleep.

[29] Chan NY, Li SX, Zhang J, Lam SP, Kwok APL, Yu MWM, et al. A Prevention Program for Insomnia in At-risk adolescents: a randomized controlled study. Pediatrics. 2021;147(3):e2020006833. 10.1542/peds.2020-006833 33627370

[30] Davidson JR, Dickson C, Han H (2019). Cognitive behavioural treatment for insomnia in primary care: a systematic review of sleep outcomes. Br J Gen Pract.

[31] Leonard JA, Duncan AB. The Effects of app-delivered cognitive behavioral therapy for Insomnia (CBT-I) on Sleep Quality, dysfunctional beliefs, and Sleep Hygiene. Psi Chi J Psychol Res. 2020;25(3):224–33.

[32] Riemann D, Perlis ML (2009). The treatments of chronic insomnia: a review of benzodiazepine receptor agonists and psychological and behavioral therapies. Sleep Med Rev.

[33] Scott H, Woods HC (2019). Understanding links between Social Media Use, Sleep and Mental Health: recent Progress and Current Challenges. Curr Sleep Med Rep.

[34] Torous J, Lipschitz J, Ng M, Firth J (2020). Dropout rates in clinical trials of smartphone apps for depressive symptoms: a systematic review and meta-analysis. J Affect Disord.

[35] Hermes ED, Merrel J, Clayton A, Morris C, Rowe M (2019). Computer-based self-help therapy: a qualitative analysis of attrition. Health Inf J.

[36] Meyerowitz-Katz G, Ravi S, Arnolda L, Feng X, Maberly G, Astell-Burt T (2020). Rates of attrition and dropout in app-based interventions for chronic disease: systematic review and Meta-analysis. J Med Internet Res.

