# Supplementary material for: Digital cognitive behavior therapy for insomnia improving sleep quality: a real-world study
Source: BMC Psychiatry. 2022 Dec 6;22:768. doi: 10.1186/s12888-022-04411-2 (PMC9727959; doi:10.1186/s12888-022-04411-2)
Supplement: Supplementary file 1 — Additional file 1. [file 12888_2022_4411_MOESM1_ESM.docx]

***Supplementary Materials*** ***for:***

**Digital cognitive behavior therapy for insomnia improving sleep quality: a real-world study**

**Supplementary Methods**

Participants were trained individually on how to use the app via a video introduction in the app by a research assistant. The components of digital cognitive behavior therapy for insomnia (dCBT-I) include sleep hygiene, sleep restriction, relaxation techniques, stimulus control and cognitive restructuring [1]. The app had videos of each training session. Homework was assigned to participants to allow time to practice these skills between sessions. Messages were automatically sent via app to remind participants to complete the tasks. The treatment effect for insomnia was reassessed via the mobile app. Participants could consult a psychiatrist via the app as necessary.

According to sleep efficiency [2, 3], sleep restriction is implemented in the first two weeks. If sleep efficiency > 90%, increase bed time by 15 minutes; if sleep efficiency between 85% and 90%, maintain the bed time; and if sleep efficiency < 85%, reduce bed time by 15 minutes. During the third and fourth weeks, stimulus control is implemented to combine with sleep restriction, if the first two-week treatment is not effective.

During the fifth and sixth weeks, if the previous stage of treatment is effective, patient education will be added. While if the treatment is ineffective, relaxation techniques is implemented to combine with stimulus control and sleep restriction. During the seventh and eighth weeks, if the previous stage of treatment is effective, the treatment will be continued. While if the treatment is ineffective, cognitive restructuring will be added. dCBT-I consisted of 8 weekly sessions, and the cognitive and behavioral components were assigned appropriately based on online self-assessment reports during 8-week to 12-week follow-up measures.

**Supplementary Results**

*Characteristics of anxiety group*

In the anxiety disorders group, 792 [30.66%] patients had medication alone, 470 [18.20%] patients had dCBT-I monotherapy, 926 [35.85%] had combined therapy (dCBT-I plus medication) and 395 [15.29%] patients reported not having treatments. See Supplementary Table 2. Compared to patients with anxiety disorders treated with medication alone and combined therapy, those treated with dCBT-I alone were younger age [Mean 43.83 years, SD 11.65] (*d* = 0.14, *P_FDR_* = 0.02; *d* = 0.18, *P_FDR_* < 0.01) with higher percentage of family history of insomnia [43.40%], lower percentage of history of using psychotropic medications [35.32%], lower GAD-7 scores (*d* = 0.32, *P_FDR_* < 0.00; *d* = 0.18, *P_FDR_* < 0.01), lower PHQ-15 scores (*d* = 0.33, *P_FDR_* < 0.00; *d* = 0.22, *P_FDR_* < 0.01), lower PHQ-9 scores (*d* = 0.40, *P_FDR_* < 0.00; *d* = 0.20, *P_FDR_* < 0.00) and lower PSQI total scores (*d* = 0.69, *P_FDR_* < 0.00; *d* = 0.51, *P_FDR_* < 0.00). In terms of the percentage of college education level and employment status, the trend was a gradual increase from medication alone group, to dCBT-I monotherapy group and combined therapy group. The percentage of long illness duration (>12 months) followed the opposite pattern.

*Characteristics of anxiety comorbid with insomnia group*

In the anxiety comorbid with insomnia group, 535 [57.34%] patients had medication alone, 45 [4.82%] patients had dCBT-I monotherapy, 120 [12.86%] had combined therapy and 233 [24.97%] patients reported not having treatments. See Supplementary Table 3. Compared to anxiety comorbid with insomnia patients treated with medication alone and combined therapy, patients treated with dCBT-I alone had higher percentage of high educational level [51.11%], lower percentage of history of using psychotropic medications [31.11%] and lower PSQI scores (*d* = 0.60, *P_FDR_* < 0.01; *d* = 0.45, *P_FDR_* < 0.01). The CBT-I alone group had younger age than the medication alone group (*d* = 0.62, *P_FDR_* < 0.01).

*Characteristics of depression group*

In the depression group, 441 [45.51%] patients had medication alone, 117 [12.08%] patients had dCBT-I monotherapy, 223 [23.01%] had combined therapy and 188 [19.40%] patients reported not having treatments. See Supplementary Table 4. Compared to depression patients treated with medication alone and combined therapy, the dCBT-I monotherapy group were of younger average age [Mean 39.19 years, SD 12.33] (*d* = 0.24, *P_FDR_* = 0.02; *d* = 0.29, *P_FDR_* = 0.02) with higher percentage of college education level [57.76%] and family history of insomnia [41.88%], more short illness duration (<3 months) [39.32%], and lower percentage of history of using psychotropic medications [39.32%] and lower PSQI scores (*d* = 0.70, *P_FDR_* < 0.00; *d* = 0.40, *P_FDR_* < 0.00). Compared to the medication alone group, the CBT-I alone group had lower PHQ-9 scores (*d* = 0.69, *P_FDR_* < 0.00), lower GAD-7 scores (*d* = 0.68, *P_FDR_* < 0.00) and lower PHQ-15 scores (*d* = 0.40, *P_FDR_* < 0.01).

*Participants in the follow-up subset*

In the 8- and 12-week follow-up subset, the insomnia group comprised 97 patients, 5 treated with medication alone, 24 treated with dCBT-I alone, 65 treated with combined therapy and 3 reported not having treatments. The anxiety group comprised 298 patients, 19 receiving medication alone, 81 receiving dCBT-I alone, 189 receiving combined therapy and 9 reported not having treatments. The anxiety comorbid with insomnia group included 46 patients, 10 receiving medication alone, 5 receiving dCBT-I alone, 27 receiving combined therapy and 4 reported not having treatments. The depression group had 68 patients, 7 treated with medication alone, 13 treated with dCBT-I alone, 46 treated with combined therapy and 2 reported not having treatments.

**Supplementary Table 1. Characteristics of insomnia group with different treatments**

| Variables | Med | dCBT-I | Med+dCBT-I | Not having treatments | Statistics |
| --- | --- | --- | --- | --- | --- |
|  | (n = 492) | (n = 260) | (n = 356) | (n = 409) | *χ² / F / H* |
| Age (years) | 46.26 (14.32) | 42.19 (11.85) | 46.32 (12.71) | 41.03 (12.25) | 17.45** |
| Sex (Female / Male) | 333 (67.68%) / 159 | 163 (62.69%) / 97 | 236 (66.29%) / 120 | 250 (61.12%) / 159 | 5.06 |
| Educational Level (low / medium / high / unknown) | 177 / 91 / 221 / 3 | 76 / 51 / 132 / 1 | 125 / 65 / 163 / 3 | 141 / 50 / 217 / 1 | 14.39 |
| Employment status (Yes / No / unknown) | 236 / 156 / 100 | 130 / 57 / 73 | 176 / 81 / 99 | 227 / 101 / 81 | 9.02* |
| Duration (< 3 months / 3 - 12 months / > 12 months) | 101 / 89 / 302 | 67 / 57 / 136 | 88 / 52 / 216 | 133 / 78 / 198 | 26.06** |
| Family history of insomnia (yes / no / unknown) | 140 / 351 / 1 | 94 / 166 / 0 | 116 / 240 / 0 | 103 / 306 / 0 | 10.83* |
| First episode (yes / no / unknown) | 98 / 382 / 12 | 52 / 160 / 48 | 73 / 253 / 30 | 93 / 312 / 4 | 1.69 |
| History of psychotropic medication (yes / no / unknown) | 343 / 148 / 1 | 66 / 194 / 0 | 241 / 115 / 0 | 100 / 309 / 0 | 302.89** |
| Impact of Life Events | 241 / 7.00 (6.00) | 31 / 6.00 (3.50) | 47 / 7.00 (4.50) | 178 / 6.00 (4.75) | 4.41 |
| PSQI | 264 / 15.00 (6.00) | 254 / 13.00 (4.00) | 347 / 16.00 (5.00) | 245 / 12.00 (6.00) | 142.72** |
| GAD-7 | 167 / 3.00 (5.00) | 222 / 3.00 (5.00) | 298 / 3.00 (4.00) | 174 / 3.00 (5.00) | 3.89 |
| PHQ-9 | 168 / 4.00 (4.00) | 223 / 4.00 (4.00) | 299 / 4.00 (5.00) | 175 / 5.00 (5.00) | 2.89 |
| PHQ-15 | 245 / 6.00 (5.00) | 251 / 5.00 (4.00) | 331 / 6.00 (5.00) | 225 / 6.00 (5.00) | 3.40 |
| ESS | 182 / 3.00 (4.00) | 225 / 4.00 (5.00) | 299 / 3.00 (4.00) | 186 / 4.00 (6.00) | 22.49** |

Mean and standard deviation (SD) for age. A total of number of subjects performed self-reported scales, and median and interquartile range (IQR) of scale scores at baseline are listed. Med, medication. dCBT-I, digital Cognitive Behavior Therapy for insomnia. For educational level, low level represented middle school education and less, medium level indicated parts of or completed high school level or high school training, and high level represented college degree and above. Impact of life events on insomnia was scored from 0 (not at all) to 10 (very serious).

PSQI, Pittsburgh Sleep Quality Index. GAD-7, Generalized Anxiety Disorder 7-Item Scale. PHQ-9, Patient Health Questionnaire-9. PHQ-15, Patient Health Questionnaire Somatic Symptom Severity Scale. ESS, Epworth Sleepiness Scale. * *P* < .05. ** *P* < .001.

**Supplementary Table 2. Characteristics of anxiety group with different treatments**

| Variables | Med | dCBT-I | Med+dCBT-I | Not having treatments | Statistics |
| --- | --- | --- | --- | --- | --- |
|  | (n = 792) | (n = 470) | (n = 926) | (n = 395) | *χ² / F / H* |
| Age (years) | 45.59 (12.87) | 43.83 (11.65) | 45.85 (11.41) | 41.86 (12.18) | 12.36** |
| Sex (Female / Male) | 580 (73.23%) / 212 | 350 (74.47%) / 120 | 702 (75.81%) / 224 | 293 (74.18%) / 102 | 1.53 |
| Educational Level (low / medium / high / unknown) | 403 / 119 / 261 / 9 | 201 / 87 / 179 / 3 | 348 / 195 / 366 / 17 | 168 / 66 / 159 / 2 | 32.77** |
| Employment status (Yes / No / unknown) | 357 / 236 / 199 | 199 / 89 / 182 | 451 / 197 / 278 | 169 / 78 / 148 | 14.34* |
| Duration (< 3 months / 3 - 12 months / > 12 months) | 219 / 122 / 451 | 116 / 80 / 274 | 213 / 166 / 547 | 140 / 62 / 193 | 23.74** |
| Family history of insomnia (yes / no / unknown) | 254 / 538 / 0 | 204 / 266 / 0 | 349 / 577 / 0 | 119 / 275 / 1 | 23.32** |
| First episode (yes / no / unknown) | 176 / 595 / 21 | 66 / 232 / 172 | 176 / 578 / 172 | 89 / 290 / 16 | 0.23 |
| History of psychotropic medication (yes / no / unknown) | 575 / 217 / 0 | 166 / 304 / 0 | 630 / 296 / 0 | 89 / 305 / 1 | 413.02** |
| Impact of Life Events | 395 / 8.00 (5.00) | 26 / 8.00 (5.50) | 89 / 7.00 (5.00) | 155 / 7.00 (4.50) | 12.23* |
| PSQI | 450 / 17.00 (6.00) | 446 / 13.00 (7.00) | 898 / 15.00 (6.00) | 271 / 14.00 (4.50) | 162.74** |
| GAD-7 | 320 / 7.00 (9.00) | 423 / 5.00 (8.00) | 811 / 6.00 (7.50) | 216 / 7.00 (7.00) | 29.49** |
| PHQ-9 | 322 / 7.00 (7.75) | 423 / 5.00 (7.00) | 814 / 7.00 (7.00) | 219 / 7.00 (6.00) | 37.55** |
| PHQ-15 | 424 / 9.00 (6.25) | 437 / 8.00 (6.00) | 875 / 9.00 (6.00) | 262 / 9.00 (6.00) | 28.13** |
| ESS | 347 / 3.00 (6.00) | 425 / 4.00 (5.00) | 831 / 3.00 (5.00) | 235 / 4.00 (5.00) | 15.02* |

Mean and standard deviation (SD) for age. A total of number of subjects performed self-reported scales, and median and interquartile range (IQR) of scale scores at baseline are listed. Med, medication. dCBT-I, digital Cognitive Behavior Therapy for insomnia. For educational level, low level represented middle school education and less, medium level indicated parts of or completed high school level or high school training, and high level represented college degree and above. Impact of life events on insomnia was scored from 0 (not at all) to 10 (very serious).

PSQI, Pittsburgh Sleep Quality Index. GAD-7, Generalized Anxiety Disorder 7-Item Scale. PHQ-9, Patient Health Questionnaire-9. PHQ-15, Patient Health Questionnaire Somatic Symptom Severity Scale. ESS, Epworth Sleepiness Scale. * *P* < .05. ** *P* < .001.

**Supplementary Table 3. Characteristics of anxiety comorbid with insomnia group with different treatments**

| Variables | Med | dCBT-I | Med+dCBT-I | Not having treatments | Statistics |
| --- | --- | --- | --- | --- | --- |
|  | (n = 535) | (n = 45) | (n = 120) | (n = 233) | *χ² / F / H* |
| Age (years) | 49.25 (10.96) | 42.40 (13.11) | 46.06 (11.56) | 42.74 (11.84) | 20.57** |
| Sex (Female / Male) | 405 (75.70%) / 130 | 31 (68.89%) / 14 | 88 (73.33%) / 32 | 183 (78.54%) / 50 | 2.48 |
| Educational Level (low / medium / high / unknown) | 263 / 88 / 182 / 2 | 15 / 7 / 23 / 0 | 41 / 30 / 48 / 1 | 82 / 31 / 120 / 0 | 30.88** |
| Employment status (Yes / No / unknown) | 289 / 237 / 9 | 28 / 12 / 5 | 63 / 41 / 16 | 133 / 87 / 13 | 5.25 |
| Duration (< 3 months / 3 - 12 months / > 12 months) | 94 / 102 / 339 | 9 / 7 / 29 | 30 / 22 / 68 | 57 / 41 / 135 | 6.87 |
| Family history of insomnia (yes / no / unknown) | 153 / 382 / 0 | 19 / 26 / 0 | 43 / 77 / 0 | 68 / 165 / 0 | 5.56 |
| First episode (yes / no / unknown) | 123 / 407 / 5 | 4 / 34 / 7 | 31 / 75 / 14 | 64 / 166 / 3 | 7.85 |
| History of psychotropic medication (yes / no / unknown) | 377 / 158 / 0 | 14 / 31 / 0 | 82 / 38 / 0 | 66 / 167 / 0 | 138.08** |
| Impact of Life Events | 402 / 7.00 (5.00) | 24 / 7.00 (6.00) | 61 / 7.00 (6.00) | 170 / 7.00 (4.00) | 2.37 |
| PSQI | 118 / 17.00 (4.00) | 45 / 14.00 (4.00) | 114 / 16.00 (5.00) | 65 / 15.00 (4.00) | 21.21** |
| GAD-7 | 25 / 6.00 (6.00) | 18 / 6.50 (8.25) | 49 / 5.00 (5.00) | 19 / 7.00 (9.00) | 4.99 |
| PHQ-9 | 26 / 7.00 (8.50) | 18 / 6.50 (9.50) | 49 / 5.00 (6.00) | 19 / 7.00 (4.50) | 2.42 |
| PHQ-15 | 103 / 8.00 (5.00) | 42 / 8.00 (5.00) | 108 / 8.00 (5.25) | 55 / 9.00 (6.00) | 4.05 |
| ESS | 41 / 3.00 (4.00) | 19 / 5.00 (5.00) | 54 / 5.00 (6.75) | 25 / 6.00 (4.00) | 4.22 |

Mean and standard deviation (SD) for age. A total of number of subjects performed self-reported scales, and median and interquartile range (IQR) of scale scores at baseline are listed. Med, medication. dCBT-I, digital Cognitive Behavior Therapy for insomnia. For educational level, low level represented middle school education and less, medium level indicated parts of or completed high school level or high school training, and high level represented college degree and above. Impact of life events on insomnia was scored from 0 (not at all) to 10 (very serious).

PSQI, Pittsburgh Sleep Quality Index. GAD-7, Generalized Anxiety Disorder 7-Item Scale. PHQ-9, Patient Health Questionnaire-9. PHQ-15, Patient Health Questionnaire Somatic Symptom Severity Scale. ESS, Epworth Sleepiness Scale. * *P* < .05. ** *P* < .001.

**Supplementary Table 4. Characteristics of depression group with different treatments**

| Variables | Med | dCBT-I | Med+dCBT-I | Not having treatments | Statistics |
| --- | --- | --- | --- | --- | --- |
|  | (n = 441) | (n = 117) | (n = 223) | (n = 188) | *χ² / F / H* |
| Age (years) | 42.82 (15.64) | 39.19 (12.33) | 42.81 (12.49) | 38.34 (14.40) | 5.93** |
| Sex (Female / Male) | 318 (72.11%) / 123 | 79 (67.52%) / 38 | 162 (72.65%) / 61 | 138 (73.40%) / 50 | 1.38 |
| Educational Level (low / medium / high / unknown) | 185 / 81 / 172 / 3 | 26 / 23 / 67 / 1 | 71 / 43 / 108 / 1 | 56 / 32 / 96 / 4 | 23.76** |
| Employment status (Yes / No / unknown) | 211 / 125 / 105 | 53 / 19 / 45 | 117 / 45 / 61 | 78 / 42 / 68 | 6.31 |
| Duration (< 3 months / 3 - 12 months / > 12 months) | 144 / 103 / 194 | 46 / 20 / 51 | 82 / 46 / 95 | 85 / 41 / 62 | 11.98 |
| Family history of insomnia (yes / no / unknown) | 129 / 312 / 0 | 49 / 68 / 0 | 82 / 141 / 0 | 63 / 124 / 1 | 8.27* |
| First episode (yes / no / unknown) | 121 / 307 / 13 | 18 / 58 / 41 | 54 / 130 / 39 | 47 / 133 / 8 | 1.18 |
| History of psychotropic medication (yes / no / unknown) | 303 / 138 / 0 | 46 / 71 / 0 | 155 / 68 / 0 | 48 / 139 / 1 | 130.56** |
| Impact of Life Events | 196 / 8.00 (5.00) | 9 / 6.00 (5.00) | 18 / 6.50 (7.00) | 70 / 8.50 (4.75) | 3.39 |
| PSQI | 294 / 17.00 (6.00) | 111 / 14.00 (7.00) | 212 / 16.00 (7.00) | 133 / 14.00 (5.00) | 63.73** |
| GAD-7 | 208 / 15.00 (10.00) | 104 / 9.50 (12.25) | 194 / 11.00 (13.00) | 96 / 15.00 (11.00) | 46.27** |
| PHQ-9 | 210 / 18.00 (9.00) | 106 / 14.00 (14.50) | 195 / 15.00 (13.50) | 96 / 18.00 (6.25) | 44.72** |
| PHQ-15 | 280 / 11.00 (7.00) | 108 / 10.00 (7.00) | 206 / 11.00 (7.00) | 123 / 12.00 (7.00) | 18.69** |
| ESS | 231 / 4.00 (9.50) | 108 / 6.00 (7.00) | 196 / 4.00 (6.00) | 105 / 6.00 (8.00) | 15.74* |

Mean and standard deviation (SD) for age. A total of number of subjects performed self-reported scales, and median and interquartile range (IQR) of scale scores at baseline are listed. Med, medication. dCBT-I, digital Cognitive Behavior Therapy for insomnia. For educational level, low level represented middle school education and less, medium level indicated parts of or completed high school level or high school training, and high level represented college degree and above. Impact of life events on insomnia was scored from 0 (not at all) to 10 (very serious).

PSQI, Pittsburgh Sleep Quality Index. GAD-7, Generalized Anxiety Disorder 7-Item Scale. PHQ-9, Patient Health Questionnaire-9. PHQ-15, Patient Health Questionnaire Somatic Symptom Severity Scale. ESS, Epworth Sleepiness Scale. * *P* < .05. ** *P* < .001.


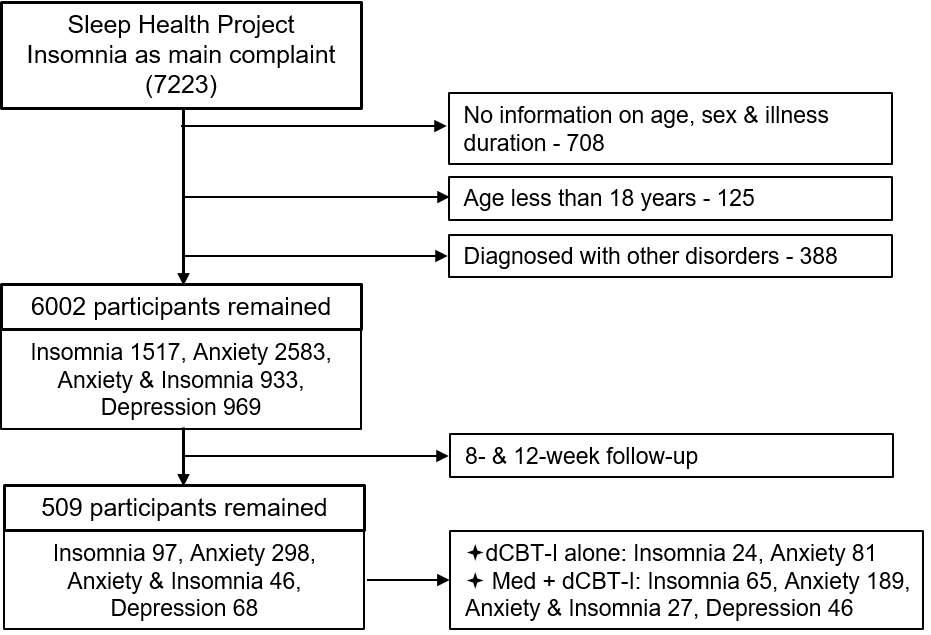


**Supplementary Fig. 1 Data screening flowchart**

Med, medication. dCBT-I, digital Cognitive Behavior Therapy for insomnia.

**References**

1. Pigeon WR: **Treatment of adult insomnia with cognitive-behavioral therapy**. *Journal of clinical psychology* 2010, **66**(11):1148-1160.

2. Buysse DJ, Reynolds CF, 3rd, Monk TH, Berman SR, Kupfer DJ: **The Pittsburgh Sleep Quality Index: a new instrument for psychiatric practice and research**. *Psychiatry research* 1989, **28**(2):193-213.

3. Reed DL, Sacco WP: **Measuring Sleep Efficiency: What Should the Denominator Be?** *Journal of clinical sleep medicine* 2016, **12**(2):263-266.
